# Cell Cultures for Virology: Usability, Advantages, and Prospects

**DOI:** 10.3390/ijms21217978

**Published:** 2020-10-27

**Authors:** Alexander A. Dolskiy, Irina V. Grishchenko, Dmitry V. Yudkin

**Affiliations:** State Research Center of Virology and Biotechnology “Vector”, Rospotrebnadzor, World-Class Genomic Research Center for Biological Safety and Technological Independence, Federal Scientific and Technical Program on the Development of Genetic Technologies, 630559 Koltsovo, Novosibirsk Region, Russia; dolskiy_aa@vector.nsc.ru (A.A.D.); grischenko_iv@vector.nsc.ru (I.V.G.)

**Keywords:** cell cultures, virus, reporter construction, virus-inducible expression, cell-based method, cell susceptibility, cell permissivity

## Abstract

Virus detection in natural and clinical samples is a complicated problem in research and diagnostics. There are different approaches for virus isolation and identification, including PCR, CRISPR/Cas technology, NGS, immunoassays, and cell-based assays. Following the development of genetic engineering methods, approaches that utilize cell cultures have become useful and informative. Molecular biology methods allow increases in the sensitivity and specificity of cell cultures for certain viruses and can be used to generate reporter cell lines. These cell lines express specific reporter proteins (e.g., GFP, luciferase, and CAT) in response to virus infection that can be detected in a laboratory setting. The development of genome editing and synthetic biology methods has given rise to new perspectives regarding the design of virus reporter systems in cell cultures. This review is aimed at describing both virology methods in general and examples of the development of cell-based methods that exist today.

## 1. Introduction

Viruses are infectious agents associated with many diseases in the human population, some of which can lead to epidemics and pandemics. To prevent such events, both the quick detection of agents isolated from patient and natural samples and the study of the properties of isolates are necessary. Currently, there are several approaches for virus detection. There are detection methods associated with PCR, CRISPR/Cas technology, NGS (next generation sequencing), immunoassays, and cell-based assays [1,2,3,4,5,6,7,8] (Table 1). All of these approaches have both advantages and limitations and must be used to solve specific problems for the comprehensive study of viruses. Some approaches allow only the detection of viral nucleic acids or proteins, while others allow the detection of viable viral particles and can be used to establish the dose and time of infection and the mechanisms of life cycle. The most widespread group of virus detection methods is based on the amplification of viral genome material. In addition to PCR-based detection, there are other methods based on amplification (e.g., SDA—strand displacement amplification [9], NASBA—nucleic acid sequence-based amplification [10], 3SR—self-sustained sequence replication [11], and LAMP—loop-mediated isothermal amplification [12]). These methods can be applied not only in research but also in clinical detection. These methods allow the direct detection of viral DNA, but in the case of viral RNA, reverse transcription must precede PCR [1,13]. PCR-based methods are sufficiently sensitive because of amplification, which allows the detection of only a few molecules in a sample. However, some authors note that the sensitivity of PCR-based methods is lower than the sensitivity of cell-based methods in the detection of certain viruses, such as human enteric virus [4]. Moreover, the use of PCR-based methods can be related to false-positive results. Additionally, PCR-based methods allow the detection of only the viral genome and not the live infectious virus. However, improved virus detection approaches that are related to next-generation sequencing are currently being developed. These methods have mainly been used to detect purified and concentrated viruses (i.e., from cell culture), and a problem in using this method for virus detection in clinical samples has been that virus-to-host genome ratios are insufficient [1,13]. To solve this problem, a method that can effectively increase the number of viral genomes relative to host genomes was devised through a detailed comparison of modern approaches for the purification and enrichment of viral particles from clinical material. This led to a reduced cost of sample preparation for sequencing as well as the improvement of subsequent bioinformatic analysis [4]. The CRISPR-Dx method for virus detection is based on guided endonucleases: CRISPR associated proteins (Cas). This method uses proteins of the Cas13 family, which exhibit “collateral,” nonspecific cleavage activity toward RNAs [14]. Considering this effect, a method designated SHERLOCK (specific high-sensitivity enzymatic reporter unlocking) was established. This nucleic acid-detection tool with high sensitivity involves viral genome material amplification and the indirect cleavage of fluorescently labeled RNA probes by the Cas13 protein, allowing the quantitation of the viral material in the sample [15]. The sensitivity of this method results from the fact that even insignificant amounts of viral nucleic acids from biological material are subjected to recombinase polymerase amplification (RPA) [16], with an additional reverse transcription step for RNA virus detection. The amplified DNA copies of the viruses are then converted back to RNA using DNA-dependent RNA polymerase T7. Thereafter, using gRNA, the Cas13 protein recognizes viral nucleotide sequences, leading to the collateral cutting of the labeled nucleotides, which allows the quantitative determination of the viral content in the samples. This direction system is continuously being developed to increase its sensitivity and improve the detection of Dengue virus or Zika virus (ZIKV) [2,3].

Another popular method for virus detection is to use antigen-based assays. This approach can be used both in research and in clinical diagnostics. The standard methods using antibodies against viruses include virus neutralization tests, hemagglutination inhibition, complement fixation, and indirect immunofluorescence. These methods are used only for diagnosing already known viruses whose capsid structure has been studied. The evaluation of viral titers using end-point dilution analysis is based on a hemagglutination test of the cell culture supernatant. However, as observed for influenza virus due to the rapid accumulation of mutations in the viral genome sequence encoding hemagglutinin leading to amino acid replacements, the investigation of the antigenic characteristics of viruses using this method is complicated [17]. One of the problems in the application of this method is the poor replicative ability of the virus in standard cell lines, such as MDCK cells [18]. Perhaps this assay will be improved with the use of new sensitive lines. In this regard, the improved monitoring of virus circulation using an antigen-based assay based on the detection of a viral nucleoprotein would facilitate the measurement of the viral titer in clinical samples [17]. Such a technique has been developed for IgG (immunoglobulin G) and IgM (immunoglobulin M) proteins, which allows the monitoring of diseases in a human population. In the case of diseases associated with flaviviruses, IgM is typically produced within several days after the manifestation of disease and can frequently be detected for a few months. However, in cases of flavivirus infection, the cross-reactivity of antibodies is possible [19]. In addition, IgG is detected within a few days after the development of IgM antibodies and can be detected for several months. Using this approach, detection methods for many viruses, such as ZIKV [5] and hepatitis E virus [20], have already been made available.

There is a group of methods for detecting viable viruses using cell culture. The development of these methods for plaque detection began in 1950 [21,22]. Methods for detecting viruses associated with cell culture have been developed and show great potential because they present a number of advantages. Using these methods, it is possible to both determine the presence of viral particles and characterize viral properties, which is extremely important when developing approaches for the treatment of new virus-induced diseases. Additionally, viral particles obtained from cell cultures can be studied by electron microscopy for the examination of viral morphology. All approaches for the identification and study of viruses using cell culture can be divided into two categories. The first approach takes advantage of the cytopathic effect (CPE) of viral infection, which means that cells die after virus release and can be detected and quantified under a microscope. However, methods based on CPE are laborious and exhibit low sensitivity, and they are not able to detect noncytopathic viruses. Different cell lines can be used for virus detection, such as NSK, MDCK, UMNSAH/DF1, BS-C-1, Vero, A549, HEK293, or HeLa cells, which can serve as substrates for the culture and detection of viruses with differences in efficiency and specificity [6,23,24]. The second approach is related to the generation of a reporter cell line, in which specific cells are modified to produce a reporter protein in response to virus infection. The properties of this reporter should be very specific to the virus to allow researchers to specifically detect the virus of interest. In the case of viral infection, the viral protein recognizes the reporter construct as a viral genome or a chimeric protein with a specific cleavage site, which generates a quantifiable signal that can be detected by researchers. There are different principles for the detection of different viruses depending on their genome structure and life cycle. The transactivation of viral structures in the vectors of some RNA viruses can occur through the infection of virus proteins, the cleavage of proteins and the release of reporters by viral proteases, and negative-strand RNA viruses with nuclear replication cycles can be detected with minigenome mimic viruses [8].

In summary, cell-based methods are one of the important categories of viral detection and characterization approaches that are currently available. All of these strategies have advantages and disadvantages, which are described in this section, but the main distinguishing characteristic is that cell-based methods can be used to isolate and study living viral particles; however, there is a problem in the application of these methods related to low susceptibility (virus ability to enter cells) and permissivity (virus ability to replicate in cells) in cell cultures. Since these two properties of cell lines make a shared contribution to the development of cellular methods, they can be generally referred to with the term “sensitivity” unless specific differences are mentioned in a report. To solve this problem, new approaches are being developed. The sensitivity of cell lines to specific viruses depends on both the virus attaching to cell membrane receptors and the host genes associated with the cellular antiviral response. Modern methods of molecular biology have allowed the development of genetically modified cells in which viral production is increased [25,26,27]. This review is aimed at describing both virology methods in general and specific examples of the development of cell-based methods that are used today.

## 2. CPE Detection and Sensitive Cell Lines

Technologies for increasing the sensitivity of cell cultures are developed to improve approaches for the study of viable viral particles. To study the biology of viruses, such as the factors determining their host species specificity and proliferation rate and the production of viable viral particles, cell lines that allow virus propagation are needed. One of the essential properties of a virus is its ability to enter the cell and to produce viral progeny. Viral replication is followed by morphological and biochemical host cell alterations, which ultimately lead to cell destruction. There are several forms of these changes depending on the virus species, such as syncytium formation, cell rounding, or cell lysis. Such visible cellular changes are commonly referred to as cytopathic effects [28]. Based on this characteristic, many assays for virus infectivity have been developed (e.g., the median tissue culture infectious dose (TCID50), half of the lethal dose (LD50), pock and plaque assays), as have other biological/chemical assays (e.g., for the detection of hemadsorption, hemagglutination, and total protein) and direct viral particle counting by electron microscopy. In the development of technologies for virus research, embryonated chicken egg (ECE) methods are an alternative to cell-based virus research techniques. For some viruses, ECEs are still often used for virus isolation. Although ECEs can support the growth of various influenza virus isolates, they have several limitations, which are related to the fact that serial reproduction in embryos can lead to the alteration of hemagglutinin glycoproteins, which results in the production of egg-adapted viruses that differ from the original isolate [29]. Studies have compared these two methods with the aid of the hemagglutination test by comparing different cell lines and using information about the CPE and TCID50/mL. It was noted that the replication efficiency of the low-pathogenicity avian influenza virus was lower in all cell lines than in embryos. However, the authors emphasized that the use of cell lines such as MDCK, CEK, and CEF cells saves time and resources by allowing the processing of a larger volume of samples, which is potentially beneficial despite the lower virus replication efficiency [30]. Cellular methods that use CPEs as one of the main tools have now also been applied to other types of viruses. For example, such methods were used to study a taterapox virus that is phylogenetically closely related to smallpox virus. Using this method allows a taterapox virus to be distinguished from closely related ectromelia and vaccinia viruses due to differences in cytopathic effects [6]. Additionally, cell-based methods were used to study the differences between several isolates of ZIKV. In these studies, the cytopathic effect on the Vero cell line was used to estimate the infectious titer as one approach for studying viral strains. It was found that two Zika virus isolates, NR-50244 and NR-50241, can effectively infect HEK293 cells, which quickly leads to a noticeable CPE and general cell lysis [24]. Modern approaches make it possible to not only search for specific cell lines but also identify the mechanism underlying the observed effect, which is associated with different cellular receptors. Therefore, research such as systematic studies of ZIKV tropism toward various cell lines has been conducted. ZIKV has been shown to exhibit tropism toward more than seven cell lines derived from different tissues, such as muscle, placenta, and liver [31]. The broad tropism can be explained by the widespread distribution of potential ZIKV receptors in various organs [32]. It was previously shown that in a human epithelial cell line, this virus replicates actively, resulting in an antiviral effect of IFN-β (interferon beta) [33]. These studies reported a broad range of cell lines that are useful for growing ZIKV in virology laboratories. From all of the above methods, we can conclude that strategies based on CPE are widely used in the study of different viruses. However, these methods are often time consuming, and it is not always easy to select a cell line that will be sensitive and ideally selective for a specific type of virus. In addition, increasing the sensitivity of cell cultures is important for developing methods for the detection of viral particles using reporter constructs that will help to improve cell-based technologies. This problem can be solved in two ways. It is possible to increase the sensitivity of cell cultures for the specific virus of interest or to create a cell line with hypersensitivity to all viruses to solve the problems related to detecting unknown viral agents from patient samples. This topic remains relevant because the use of several standard cell lines is sometimes required to detect viruses. If a virus-specific cell line is not used, then more than one cell line should be used for virus detection. For instance, in experiments involving respiratory virus isolation from 16 samples, including influenza samples, 3 samples showed CPEs only in Mv1 Lu cells; 3 showed CPEs only in MDCK cells; and the remaining samples showed CPEs in both cell lines [27]. There are already a number of examples of increased susceptibility and permissivity of cell cultures to certain viruses. For example, human influenza virus hemagglutinin interacts with alpha 2-6 sialic acid, while avian influenza virus hemagglutinin exhibits specificity to alpha 2-3 sialic acid [34], which was taken into account in the development cell lines sensitive to influenza virus. The open reading frame of SIAT1 (CMP-N-acetylneuraminate-beta-galactoside-alpha-2-3-sialyltransferase) was cloned into a plasmid, and the MDCK cell line was transfected. The obtained cell line, MDCK-SIAT1, showed higher sensitivity to human influenza virus than to avian influenza virus [26]. Approaches for increasing the expression of enzymes involved in the synthesis of cell surface receptors are promising to create sensitive cell lines for different viruses. For example, there is no appropriate propagation system for norovirus (HuNoV) currently existing [35]. Nevertheless, it has been known that HuNoV interacts with receptors belonging to the histo-blood group antigens located in cell membrane [36]. Maturation of these receptors is a multi-stage process. However, there is evidence of the involvement of enzymes such as specific glycosyltransferases in these pathways [37]. Therefore, overexpression of these enzymes may possibly increase the sensitivity of cell cultures to norovirus.

In another study related to the influenza virus, the MDCK cell line was modified by the overexpression of human airway transmembrane proteases (TMPRSS2 or MSPL). In the absence of trypsin, these cells supported the growth of three recombinant vaccines based on pathogenic avian influenza viruses [38]. The following studies presented as examples were associated with increased permissivity of cell lines to a widespread dsDNA genome virus—herpes simplex virus (HSV). One of the first sensitive cell lines was produced for HSV detection. A construct containing the viral trans-activator protein VP16 (known as α-TIF or Vmw65 [39]) with the long terminal repeat (LTR) sequence of Moloney leukemia virus was produced for cell transfection. Producing VP16 makes cells more sensitive to HSV and increases virus production 200-fold, which is convenient for a CPE assay [40]. To create reporter cell lines for virology studies, it is necessary to increase both susceptibility and the replication rate of the viral genome in the cell. Having solved this problem, it will be possible to both generate high-resolution reporter cell lines and increase the production of vaccine strains. It has been shown using RNA interference that a decrease in the activity of certain genes in mammalian cells associated with the immune response leads to increases in EV71 (poliovirus and enterovirus 71) growth by up to 80-fold [27]. It has been found that the yield of viral particles can be increased by the knockdown of genes such as EP300 (histone acetyltransferase p300) and ZNF205 (zinc finger protein 205) by using CRISPR gene editing technology. A similar study described 18-fold increases in rotavirus growth achieved through the knockout of a single Vero cell host gene [25]. Promising results have been obtained by knocking out the BST-2 (bone marrow stromal antigen 2) gene, thereby blocking viral release from cells. The exit of some viruses with lipid envelopes, such as MHV68 (murine gamma herpesvirus 68), porcine epidemic diarrhea virus, influenza virus, and vaccinia virus, was shown to be significantly increased in BST-2-knockout animal cell lines [41]. The results of this study showed that this approach can increase the production of many viruses. The replication of many viruses is associated with cell destruction. For example, members of the Adenoviridae family initiate cell death through a p53-independent mechanism [42]. In this regard, an interesting approach for increasing the sensitivity of cell cultures is to generate apoptosis-resistant cell cultures. Apoptosis is a multicomponent mechanism that engages caspases or AIF1 (allograft inflammatory factor 1) in the caspase-independent pathway. Knocking out all of these genes is necessary to inhibit this mechanism. This problem was solved with the help of CRISPR/Cas9 technology, where the use of several sgRNAs led to the removal of these genes from the HEK293T cell line. Further analysis showed that viral particles that carry proapoptotic proteins were produced more efficiently than in a standard cell line [43].

It can be concluded that virus isolation by cell culture followed by CPE determination is the most commonly used method for virus identification in biological samples [44], and modern approaches for improving the sensitivity of cell cultures are very promising. Nevertheless, it is worth discussing the shortcomings of this approach that exist today. The quantification of plaque-forming units (PFUs) is a widely performed CPE-based approach for quantifying the levels of different viruses, but this method is laborious; for example, most virus families replicate for a long time (up to week), and cell culture takes place under specific conditions (humidity, pH of medium). In addition, the cultivation of viruses requires certain safety precautions that are necessary when working with pathogenic agents. Moreover, the majority of standard virus detection protocols include cell fixation with immunostaining in the days after infection [8,44]. Additionally, this approach is based on using suitable cell lines and the application of other methods for detecting viruses, such as the detection of viruses using antibodies. Since low viral particle counts often occur in a sample, antigen-detecting antibodies are not always available, and the isolation and production of viral particles requires many attempts and the need to develop cell lines with increased sensitivity to virus infection. Consequently, this method does not provide rapid results. In addition, some viruses (i.e., Lassa virus) show very low PFU values, and the application of the CPE method is therefore not useful [45]. Even viruses that are relatively easy to grow in a wide range of cell lines can be time consuming and expensive to culture.

## 3. Reporter Cell Lines

To make cell culture-based methods simpler, more informative, and more convenient, reporter cell lines have been developed. A reporter cell line comprises specific cells that produce a reporter protein in response to virus infection. The reporter should be very specific to the virus of interest to allow researchers to detect a certain virus. Upon viral infection, a viral protein recognizes a reporter construct as a viral genome or chimeric protein and produces a signal that can be detected. Reporter cell lines can be very helpful for virological studies and vaccine manufacture. Although reporter-based methods can require the growth live pathogenic viruses and therefore be confined to laboratories with certain biosafety levels, they are easy to scale up and less laborious than standard virological methods, thus greatly increasing infectious virus detection. Such methods have led to rapid, objective virus quantitation studies, including the assessment of viral titers, antiviral susceptibility, and neutralization titers [7,46]. The use of reporter cell lines presents a number of considerable advantages over classical cell-based techniques. First, the method is faster than the other methods based on viable viruses. Under this approach, time-consuming culture and immunostaining techniques are not required, and the results can be obtained in 12–48 h [8,47]. Another advantage is that the reporter cell lines can detect a small number of infected cells. For example, reporter cell lines showing luciferase activity for the varicella-zoster virus can be used to detect fewer than 10 infected cells per sample [48].

The key feature of a reporter construct is the reporter gene. Fluorescent proteins and enzymes such as beta-galactosidase or luciferase can serve as reporter genes. It should be noted that there are various elements used for detecting reporter activity in studies of different virus families depending on their genome structure and life cycle. Negative-strand RNA viruses (orthomyxoviruses, filoviruses) showing replication in the host nucleus have been detected using a construct known as a minigenome [7,8]. The reporter system for viruses with an (+)RNA genome (alphavirus or hepacivirus) also involves a minigenome construct that can be recognized by viral proteins [46,49]. However, a different approach is used for this group of viruses. Some viruses with an RNA(+) genome (flaviviruses [50,51] and enteroviruses [52]) encode a large polypeptide that includes structural and nonstructural proteins. This polypeptide is further processed into individual proteins by host and viral proteases. Virus infection can be detected when the reporter is activated in response to the cleavage of a specific site at the peptide by viral proteases. Retroviruses and viruses with DNA genomes (herpesviruses) can be detected after stable cell transfection by plasmids with reporter constructs regulated by viral promoters [47,53]. The transcription of the reporter cassette is initiated by virus entry.

Below, we will consider the development of some reporter cell lines that are currently used in virology for the detection of different virus families. These studies can provide a basis for the development of detection systems for novel viruses based on similarity of the design approaches used to assemble the reporter constructs for previously characterized viruses belonging to the same class.

### 3.1. Reporter Cell Lines for ssRNA(−) Genome Virus Detection

#### 3.1.1. Orthomyxoviridae, Influenza Viruses

**Figure 1:**
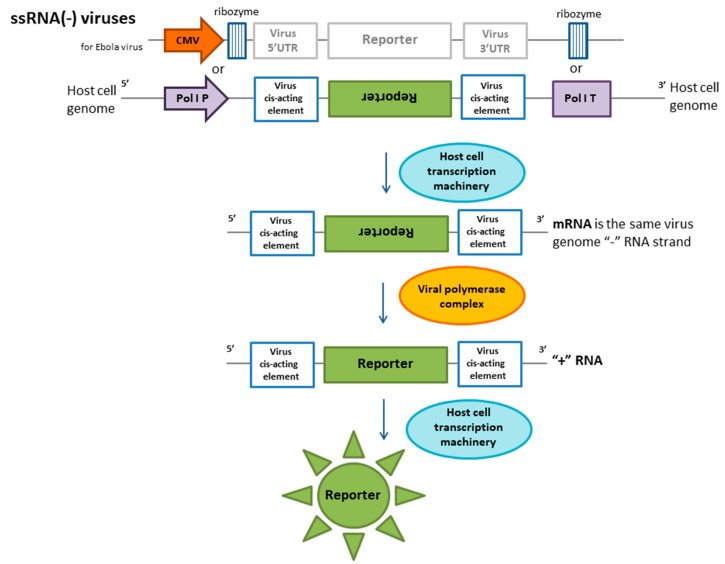
Molecular reporter system for ssRNA (−) genome virus detection. The principle of the reporter system is based on the production of mRNA without the cap and the polyA tail. Such RNAs mimic the virus genome. Ribozymes hydrolyze RNA or RNA-polymerase I promoters. This transcript mimics (−) viral RNA, which allows it to be replicated using viral proteins. The transcription of the reporter protein occurs from (+) RNA obtained in this manner.

Influenza A and B are widespread viruses that are responsible for annual epidemics in humans and are associated with large economic losses. Influenza is categorized into different subtypes and strains depending on its neuraminidase and hemagglutinin types [54]. The accurate and rapid detection of these viruses in biological samples is crucial for virus infection diagnostics and vaccine manufacturing. The reporter cell line concept was first applied to influenza viruses [7]. Several years later, reporter cassettes for influenza A and B viruses were developed by Li et al. Influenza virus-activated reporter cell line techniques for research include the generation of a reporter system that can produce any signal in response to virus replication. The genomes of influenza A and B viruses are divided into eight segments [55]. In the host cell nucleus during infection, proteins of the viral polymerase complex and the NP nucleoprotein are involved in the production of RNA(+) chains and viral genome replication [56]. To activate these processes, cis-acting elements are required, which are nontranslated conserved regions at the ends of the genomic influenza virus RNA sequence (5′- and 3′-UTRs) [57]. The reporter construct for influenza virus A used in cells contains a luciferase- or GFP (green fluorescent protein)-encoding region located between the 5′- and 3′-UTRs of the NP genomic segment. This construct is cloned into plasmid DNA (pDNA) and driven by polymerase I promoter and terminator elements, which generates RNA with no additional nucleotides at the 5′- or 3′-end. The linearized pDNA integrates into the host cell genome (Figure 1). This construct mimics a virus genome segment during infection, and reporter gene expression is achieved using the viral polymerase complex. A reporter system for the influenza B virus was generated via a similar approach. Reporter cell lines were obtained from HEK293T, BHK-21, and MDCK cells [7,55]. Using a set of respiratory viruses, the activation specificity of the reporter protein was evaluated. Specific induction was high, with a slight cross-activation between influenza A and B viruses [55,58]. Although the determined TCID50 was similar in the reporter cell lines and MDCK cells, there were clear advantages of the reporter lines in terms of a reduced time to obtain results (up to two days instead of six days), enhanced objectiveness, and decreases in laborious experimental procedures. These reporter cell lines were successfully used in antibody neutralization and antiviral susceptibility experiments.

#### 3.1.2. Filoviridae, Ebolavirus

Ebolavirus is a filovirus that causes hemorrhagic fever that has been widespread in Central Africa and is now emerging in Western Africa and Europe. Ebolavirus disease takes the form of an acute fever and results in approximately 50% mortality [59]. As mentioned, molecular methods are very useful, but the results do not permit the detection of infectious viruses. This makes it difficult to assess the pathogenicity of a particular viral isolate and predict outbreaks, which are of great importance for ebolavirus studies. The standard cell-based method also presents limitations in ebolavirus studies, including the 14-day period before results are obtained. All of these characteristics are key prerequisites for the development of reporter cell lines.

Reporter cell lines for ebolavirus detection were developed based on the negative-sense minigenome, which is regulated by the constitutive cytomegalovirus (CMV) promoter for transcription by cellular RNA polymerase II [8] (Figure 1). The addition of hammerhead ribozyme (corresponding to ribozyme A with some modifications) causes the mRNA to mimic the ebolavirus genome. The target transcript is therefore not capped or polyadenylated. Thus, a chimeric minigenome was assembled that consisted of the following sequentially positioned functional components: Minimal viral promoter, 5′-UTR of ebolavirus RNA, green fluorescent protein sequence, and 3′-UTR of viral RNA. This construct was flanked by two ribozymes. The constructed cassette was integrated into the VERO-E6 cell line genome using the PiggiBac transposon. The transcription of the minigenome was mediated by the viral replication complex, producing a reporter protein. This system is effective for different species of ebolavirus without cross reactivity with closely related Marburg viruses. A comparison of this cell-based approach with an immunofluorescence assay for virus quantification showed that they presented the same efficiency [8]. Additionally, clinical samples were tested, and the assay produced positive results in reporter cells.

### 3.2. Reporter Cell Lines for ssRNA(+) Genome Virus Detection

#### 3.2.1. Togaviridae, Alphavirus

**Figure 2:**
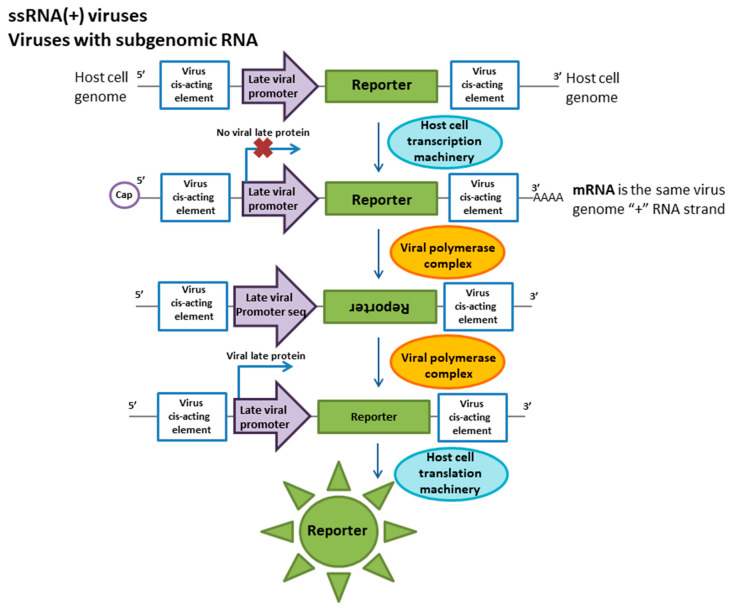
Molecular reporter system for ssRNA (+) genome viruses with subgenomic RNA detection. The reporter system scheme is based on the capability of such viruses to synthesize structural proteins from subgenomic (+) RNA as a product of virus replication. In the first stage, mRNA mimicking the virus (+) RNA will be produced in the cell. During infection, the (+) RNA is recognized by viral proteins and replicates to form the (−) RNA copy. In the next step, this (−) RNA serves as a matrix strand for the transcription of subgenomic (+) RNA; the late promoter is activated by viral proteins, and the reporter protein can be synthesized.

The subfamily of alphaviruses includes the Sindbis virus, which causes severe fever in humans. The nonsegmented genome of the virus consists of two functional regions. The direct translation of the polyprotein, which is further processed by the viral protease, begins from the first part of the ssRNA(+). These proteins are required for the transcription of late proteins and the replication of the viral genome. Late or structural proteins are encoded by subgenomic RNA transcribed from ssRNA(−). The cDNA cloning of the Sindbis virus genome was the first step toward reporter cell line development [49]. The reporter construct was based on the cDNA of the Sindbis virus subgenomic RNA [60]. The reporter cassette is under the control of a Rous sarcoma virus long terminal repeat (LTR) promoter cloned into a plasmid and can be constitutively expressed in a cell. This transcript mimics ssRNA(+) and consists of a late promoter and a firefly luciferase ORF flanked by viral cis-acting elements (Figure 2). When a cell is infected by the virus, nonstructural viral proteins that recognize cis-elements are produced, and ssRNA(−) is synthesized. This RNA is a template for the synthesis of subgenomic RNA. These processes mimic virus life cycle stages. The translation of luciferase begins from the late promoter of the artificial subgenomic RNA. Plasmids expressing this reporter cassette were transferred into BHK cells, and stable transformants with resistance to G418 were obtained. Under a high level of Sindbis virus replication, reporter protein activity was detected for up to 4 h. Additionally, it should be noted that the luciferase assay was equivalent to and as sensitive as CPE assays. However, luciferase activity was detected 26 h after infection when no CPE was observed. Although the Sindbis virus is not considered a serious human pathogen, other viruses that are both more closely related to alphaviruses (eastern/western equine encephalitis virus) and more distantly related (rubella virus) are important pathogens, and there is great interest in developing specific and accurate techniques for detecting these agents [49].

#### 3.2.2. Togaviridae, Rubivirus

Rubella is a severe contagious viral infection. Although the disease caused by this virus is mostly mild in children and adults, during pregnancy, it can lead to stillbirth or fetal malformations. Thus, an accurate diagnosis of rubella is necessary when infection is suspected [61,62]. Currently, the diagnosis of rubella pathogens is based on serological methods and reverse transcription PCR (RT-PCR) and PCR-based methods [61,63]. However, the isolation of a viable virus for further studies in cell cultures is a very laborious approach since rubella does not have a marked cytopathic effect on cell cultures. Thus, the detection method for this virus is based on coinfection by rubella and an enterovirus (e.g., Coxsackie virus, a member of the echoviruses) with an obvious CPE for 10 days [64]. When these viruses are cocultivated, the cytopathic effect and the replication of the enterovirus decreases in response to the production of interferon induced by the rubella virus, which indicates the presence of this pathogen in the host cells. A cell line with a specific reporter for rubella virus has been developed to facilitate studies using cell-based methods without adding helper viruses. As in other togaviruses, the rubella virus genome consists of ssRNA (+) and is divided into two parts [65]. The first part encodes early proteins required for viral RNA replication, and the second encodes the structural proteins of the virion. The reporter construct is based on genetically engineered subgenomic RNA (Figure 2). This RNA carries a 600 bp deletion in the field of non-structural proteins to prevent reporter synthesis in the absence of wild-type virus. The region of structural proteins was is completely replaced by a reporter protein (chloramphenicol acetyltransferase (CAT) or green fluorescent protein (GFP)). ssRNA(–) transcribed into subgenomic RNA has been synthesized in vitro and transfected into the VERO cell line [66]. Reporter protein expression occurs only in response to infection with specific viruses. This method has proven to be sensitive to both laboratory strains and viruses from clinical samples [67].

#### 3.2.3. Flaviviridae, Hepacivirus

**Figure 3:**
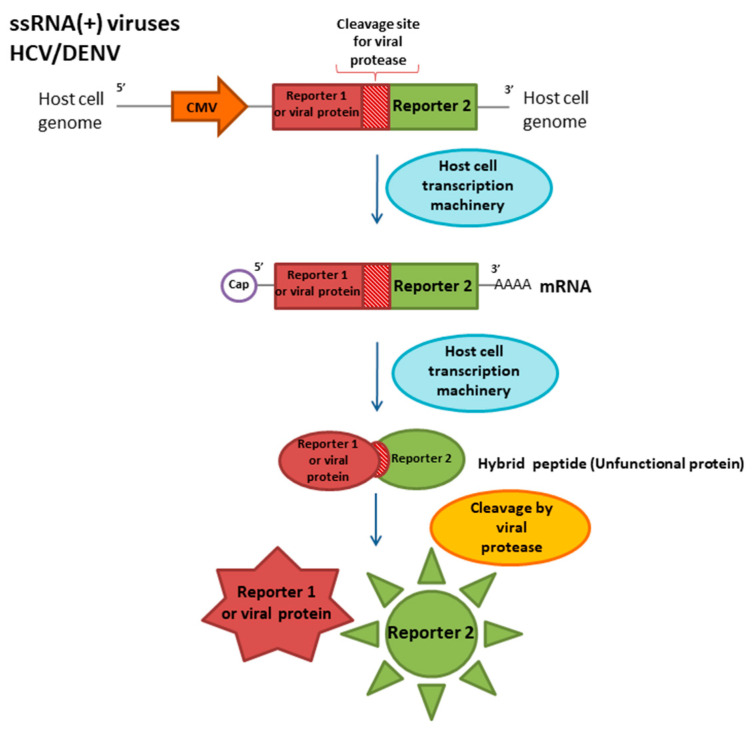
Molecular reporter system for ssRNA (+) genome viruses with genomic polyprotein detection. The reporter system is based on proteolytic cleavage by a specific viral protease in the processing of the primary viral polypeptide to generate the final proteins. The reporter protein binds to the viral protein at the cleavage sites of the viral protease and is released after infection.

Hepatitis C virus (HCV) causes severe acute or chronic hepatic injuries. HCV infections progress to chronic hepatitis, which is highly associated with hepatocellular carcinoma. An estimated 71 million people exhibit chronic hepatitis C virus infection [68]. Hepatitis C is detected in clinical samples using PCR-based and immunologic methods, providing sufficient diagnostic precision. However, to study the nature of a viable virus, methods based on continuous cell lines are required. Since the hepatitis C virus is difficult to reproduce in standard cell cultures, various approaches based on genetically engineered constructs and cell lines have been developed that simulate different virus life cycle stages [51]. One such approach is illustrated by a cell line with a reporter cassette that is activated in response to virus entry and infection. Moreover, it is necessary to develop a new cell-based approach for HCV studies and antiviral drug development studies. A number of reporter systems for HCV are based on specific viral protease activity [51,69,70]. The virus genome is a sequence translated into a polyprotein. To process mature viral proteins, the polyprotein is cleaved by viral and cellular proteases. A description of the developed dual reporter system for hepatitis C virus is provided below. The reporter construct driven by the human cytomegalovirus (CMV) promoter consists of a green fluorescent protein (GFP) linked with secreted alkaline phosphatase (SEAP) via an NS4A/B junction site for the HCV NS3/4A protease. The developed reporter system delivered into hepatocarcinoma Huh-7 cells and Ava5 cells derived from Huh-7 cells using lentiviral transduction. After several weeks, stable transformants are selected. During viral infection, the HCV NS3/4A protease is expressed by the virus and separate reporter proteins, which are detected (Figure 3). The presence of both proteins was confirmed by fluorescence detection, phosphatase activity analysis, and western blot analysis. In other studies, the SEAP gene has been replaced with the luciferase gene [46,71,72].

**Figure 4:**
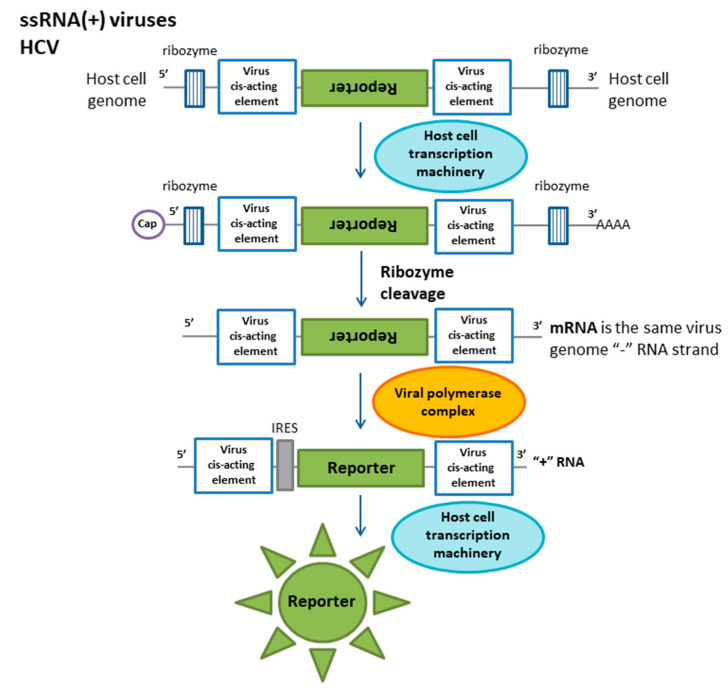
Molecular reporter system mimicking the genome for Hepatitis C virus (HCV) detection. After the transcription of the mRNA, which carries all the elements of the reporter construct, the terminal fragments are self-pinched off by ribozymes. Such mRNAs mimic the (−) RNA viral genome. Viral proteins recognize this RNA and replicate it during infection. On the (+) RNA chain, there is an internal ribosome entry site for reporter protein synthesis.

The other reporter cell-based method involves the construction of a minigenome mimicking the viral genome that is recognized by NS5B RNA-dependent RNA-polymerase (RdRP), which is a common target antiviral therapy [46]. The reporter construct is under the control of the CMV promoter. The reporter system mimics viral ssRNA(−) and consists of a 5′ viral UTR with an IRES sequence, a 3′ viral UTR and luciferase in the antisense orientation flanked by ribozymes for cleavage from host cell-synthesized mRNA (Figure 4). During HCV infection, NS5B RNA polymerase recognizes untranslated regions in the minigenomic construct without ribozymes and synthesizes ssRNA(+), which is translated in an IRES-dependent manner into the luciferase protein. This system has been transferred into BHK-21 and Ava5 cells and used for the evaluation of RdRp activity and the investigation of RdRp inhibitor effectivity. Thus, the HCV reporter cell line can be a helpful tool for studies of the nature of HCV and the search for novel compounds for HCV therapy.

#### 3.2.4. Flaviviridae, Flavivirus

Dengue virus (DENV) and Zika virus ZIKV are dangerous human pathogens capable of causing massive outbreaks [73]. The mechanism of Dengue infection is not fully understood. It is necessary to study the effects of ZIKV and DENV infection on cellular processes to predict the severity of the disease and develop targeted and effective therapies. Laboratory studies of these infections present limitations similar to those for other virus families. The cellular response to DENV infection is often detected using immunoassays or amplification-based approaches. For example, an immunostaining procedure for DENV in an infected cell culture has been developed. However, since DENV proteins are not exposed on the cellular surface, the prefixation and permeabilization of cells are required to accurately detect virus infection; therefore, this method does not make it possible to observe infections in living cells [74]. Similarly, basic methods for the identification of ZIKV infection in cells rely on immunostaining procedures with fixation and permeabilization. The limitations of the use of amplification-based methods and sequencing are the same as for other viruses and do not allow us to conduct studies with a viable virus. To observe DENV infection in real time, a Vero cell line with a reporter construct based on viral protease activity was developed. The reporter system was highly similar to the protease-induced reporter cassette for the hepatitis C virus (Figure 3); however, the reporter protein was linked to part of the viral protein NS4B through the NS3 viral serine protease recognition site. The obtained cell line was infected by four DENV serotypes. GFP expression and, accordingly, the onset of a viral infection was detected. Thus, DENV infection can be detected without cell destruction by using this reporter. However, there is a problem with this approach: The fragment of NS4B encoded in the reporter cell line has been reported to affect cellular function (e.g., to inhibit type I IFN signaling) [75]. A similar approach has been used to study ZIKV infection [76]. The viral NS4B protein coding sequence and the viral protease cleavage site were cloned in the same open reading frame with green fluorescent protein under the control of a constitutive promoter. Hepatocarcinoma cells (Huh7 cell line) and adenocarcinoma cells (A549 cell line) were transduced by lentivirus particles expressing the reporter. In infected cells producing Zika virus, the viral proteases NS2B and NS3 interact with their cleavage site, and the expression of GFP can be detected using fluorescence microscopy. The described reporter system can detect African and Asian strains of ZIKV, but the same construct is not activated by DENV. This system is very important for studies of ZIKV infection.

#### 3.2.5. Picornaviridae, Enterovirus

Enterovirus is a genus that includes many species of viruses, such as Coxsackie A and B viruses, echovirus (enterovirus A and B), poliovirus (enterovirus C), and enterovirus 68 or 72. These viruses are the most pervasive human viral pathogens [77]. Enteroviruses are the cause of many severe human diseases, such as aseptic meningitis, encephalitis, myocarditis, pericarditis, and hand, foot, and mouth disease (HFMD) [78,79]. The contamination of the aquatic environment with enterovirus is the main cause of enterovirus transmission because this virus is very stable in water and exhibits a low infectious dose [80]. Massive outbreaks of enterovirus infections have made it necessary to develop a rapid and reliable virus monitoring system.

Classical enterovirus detection and quantification approaches are based on CPE observation in cell cultures. However, this type of assay is very laborious and cannot produce rapid results; therefore, it is not convenient for routine applications. Molecular methods relying on reverse transcription PCR are much more rapid and accurate for the detection of viral genome equivalents. The limitation of this method is that it is considered to provide an indirect assessment of virus infectious ability [52].

The development of a cell-based reporter system represents a compromise between rapid experimental procedures and studies revealing the viral nature and the details of viral infection. Enteroviruses as well as other viruses with an ssRNA (+) genome produce specific proteases necessary for mature viral proteins processing [81]. One of these proteases is the highly selective and efficient protease 2A (2Apro) [82]. Thus, reporter constructs for enteroviruses are based on the specific cleavage of a fusion protein using this protease (Figure 3). This system uses fluorescence detection via fluorescence resonance energy transfer (FRET) between the acceptor fluorescent protein EYFP (enhanced yellow fluorescent protein) and the donor ECFP (enhanced cyan fluorescent protein) linked by a short peptide sequence containing the 2Apro cleavage site [52]. The cassette encoding the donor and acceptor proteins driven by a constitutive eukaryotic promoter was cloned into an expression plasmid. BGMK cells were transfected with the constructed plasmid expressing the reporter system, and stable transformants with G418 resistance were obtained. During enterovirus infection, 2Apro was expressed and cleaved its own specific site. Thereafter, interaction between the two fluorescent proteins was disrupted, resulting in an increase in the ECFP fluorescence intensity. Thus, low amounts of viral pathogens could be detected approximately 7.5 h after infection [52].

The obtained reporter cell line showed ECFP fluorescence in response to infection by three enteroviruses and was not activated by hepatitis A virus (HAV), which does not produce 2Apro. Presumably, such a reporter system can be used in research devoted to the development of antiviral drugs based on antiprotease activity.

### 3.3. Reporter Cell Lines for Retrovirus Detection

#### Retroviridae, Lentivirus

The main infectious agents in the lentivirus genus are bovine immunodeficiency virus (BIV) and human immunodeficiency virus (HIV). HIV causes acquired immunodeficiency syndrome (AIDS) [83]. AIDS leads to progressive dysfunction of the immune system and mediates the development of severe infections and multiple organ dysfunctions. BIV is a lentivirus with key genetic and structural features similar to HIV [84]. Additionally, BIV is the cause of pathological processes associated with immune system dysfunction in infected cattle.

The ability to determine the number of infectious particles in a viral stock is essential for studying viral life cycle stages, developing viral drug therapy, and searching for mutations in the virus genome. Quantitative titers of HIV stocks can be determined from end-point dilutions of the virus, but these assays are labor-intensive for a large number of samples. Therefore, investigations focused on reporter cell line development have been performed [85,86].

**Figure 5:**
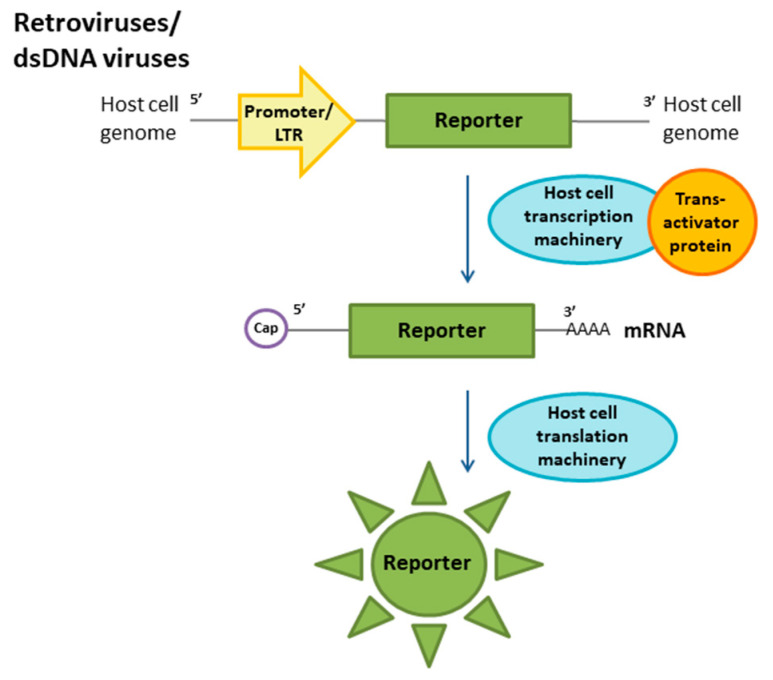
Molecular reporter system for retrovirus and dsDNA genome virus detection. In noninfected cells, there is no mRNA synthesis from the viral promoter. After the infection of the cell occurs, the transcription and translation of the reporter protein begin in the presence of the viral protein.

In HIV research, cell susceptibility is conferred by the presence of CD4 (cluster of differentiation 4) receptors on the cell surface. The first reporter cell line for HIV was developed in the late 1980s and was based on transactivation in transfected lymphoid cell lines. The CAT reporter gene was cloned under the control of the LTR promoter of HIV-1 and can be induced by the HIV Tat transactivator (Figure 5). These reporter cells were activated by HIV and, to a much lesser extent, by human T-cell lymphotropic virus type 4 and simian immunodeficiency virus (SIV). The reporter cell line was not activated by equine infectious anemia virus, human T-cell lymphotropic virus types 1 and 2, or herpes simplex virus 1 (HSV1) [85,86]. Another cell line harboring the beta-galactosidase gene under the control of the LTR promoter as a reporter for HIV detection relied on HeLa cells [53]. The same approaches were used to produce HIV reporter cell lines from human glioblastoma and rhesus macaque mammary tumors [87,88]. Thereafter, GFP driven by the HIV LTR was used to generate modified HeLa CD4 cells for HIV-1 infection detection [89].

However, an HIV LTR-based reporter system presents some disadvantages. First, this type of system exhibits a certain basal expression level in the absence of Tat. Second, it can be nonspecifically activated by cellular transcription factors, cytokines, mitogens, or even viral envelope proteins [90,91]. It was proposed that these problems could be solved by using another regulatory protein, Rev, as a transactivator. A new construct containing GFP was generated [92,93]. The advantage of using the fluorescent protein reporter system is its simplicity and the capacity to detect and infect living cells using widely available instruments. While the GFP fluorescence signal is stable under formaldehyde fixation [89], it is just as readily detected in unfixed living cells. GFP detection can be carried out with conventional fluorescence microscopy using fluorescein filter combinations. Furthermore, living cells expressing the GFP reporter can be counted and sorted by conventional flow cytometry.

A BIV detection method based on RT-PCR has been introduced [94]. Although this approach for viral detection is sensitive and rapid, it cannot be used to evaluate the infectious viral titer, which is a considerable limitation of this method. Therefore, the same approach applied for HIV was used to produce a reporter cell line for BIV. The reporter cell lines were based on bovine, canine, and lapine tissue cultures that could be infected by BIV. The CAT gene was used as the reporter gene, driven by the LTR promoter of BIV. In bovine and canine cell lines with detectable basal expression of the CAT gene, cell factors played a role in LTR expression. However, infection led to increased expression (50- to 80-fold). Bovine leukemia virus or bovine syncytial virus led to only twofold to threefold increases in expression [95]. In the further development of BIV reporter cell lines, researchers switched to GFP as the reporter gene, still driven by the BIV LTR. The obtained cell line functions as a reporter line for a very closely related virus, Jembrana disease virus, but does not function as a reporter for bovine foamy virus [96].

Another retrovirus that is currently being studied with a cell-line-based approach is simian foamy virus (SFV). A reporter cell line for SFV was constructed on the basis of BHK-21 cells. A reporter cell line with the beta-galactosidase gene driven by the LTR SFV was obtained. In this study, several SFV strains were used as laboratory-adapted strains, and natural strains from the blood of two chimpanzees, two gorillas, and one Cercopithecus individual were also used. Reporter gene expression was initiated within 72 h after infection by an autologous strain. The same result was obtained with another strain of SFV. The cells were shown to be an effective tool for the quantification of different strains. The authors showed that the replication rate of different strains of SFV is different among different cells [97].

### 3.4. Viruses With a dsDNA Genome

#### 3.4.1. Herpesviridae, Simplex Virus

Human infections involving HSV type 1 and type 2 are common throughout the world. Morbidity from herpesvirus infection is an important issue [98]. HSV infection can cause a spectrum of disorders and change the immune status of the infected person. Furthermore, HSV infection in patients with weakened immunity (AIDS patients, organ transplant recipients, leukemia patients) leads to severe diseases [47].

Although HSV is well propagated in mammalian cell cultures, the detection of virus infection is a laborious procedure, since the immunostaining of infected cells for antigen identification must be performed for accurate diagnosis. Using this approach, a result can be obtained in 24 h to 48 h in the majority of cases [99,100].

A reporter cell line allowing the more rapid detection of this virus was developed in the early 1990s [47]. The reporter systems for dsDNA viruses are similar to the reporter constructs for retroviruses (Figure 5). In this cassette, the Escherichia coli LacZ gene was used as a reporter gene, cloned under the control of the HSV ICP6 (HSV ribonucleotide reductase large subunit) promoter into an expression eukaryotic vector. Then, BHK cells were transformed with the designed reporter construct and a plasmid carrying the neomycin resistance gene. To obtain a stable reporter cell line, BHK cells were cultured with a selective antibiotic in the medium. The convenience of the reporter system involving this viral promoter arises from the fact the HSV ICP6 promoter is not activated in uninfected cells and the activation by HSV is very specific; thus, it is easy to distinguish infected and uninfected cells in the same culture. Using the described reporter system, infection detection via beta-galactosidase activity occurs within several hours (up to 6 h) after inoculation. The effectiveness of virus detection by using this reporter cell line was comparable with that of standard cell-based methods employed in virological studies, such as plaque assays. Both types of HSV activate the developed reporter cassette, whereas nonspecific activation by other members of Herpesviridae (human cytomegalovirus (HCMV), varicella zoster virus (VZV)) does not occur. Thus, an indicator cell line can provide a useful approach for the rapid observation of HSV infection in cell culture.

#### 3.4.2. Herpesviridae, Gammaherpesvirus

One of the most important species of gammaherpesviruses is Kaposi’s sarcoma-associated herpesvirus (KSHV or human herpesvirus 8 (HHV8)). This virus can cause Kaposi’s sarcoma, a cancer that often develops in AIDS patients. A barrier to studying viral life cycle features by cell-based methods is related to the fact that the virus is difficult to cultivate in most cell lines. Therefore, the use of such standard methods as determining the titer of the virus is challenging. Thus, the development of reporter cell lines is based on cells that are permissive, at least at a low level (HEK293T cells, immortalized endothelial cells, some carcinoma cell lines, etc.) [101]. As noted previously, molecular biology-based methods based on PCR- or ELISA-based genome equivalent measurements do not reflect virus infectious ability.

For indicator cell line development the following approach was used. The HH8 genome encodes the major regulator Rta, which activates a range of host and viral promoters and regulates lytic program activation in the viral life cycle [102,103,104]. One of the promoters regulated by Rta is the cellular promoter of polyadenylated nuclear (PAN) RNA. A fragment of this promoter that can be activated by the wild-type virus and the lacZ gene were cloned into a reporter plasmid. Then, HEK293T cells were stably transfected. It was shown that infection by HHV8 virus activated the reporter cassette and that the level of beta-galactosidase activity was virus dose dependent.

#### 3.4.3. Herpesviridae, Varicellovirus

Varicella-zoster virus (VZV) is a cause of varicella, which may present severe complications due to primary infection or virus reactivation in infected cells [105]. The available, rapid, well-targeted approaches that allow the detection of VZV are time consuming and labor-intensive, which makes the development of targeted viral therapies and drug-resistant strain identification complicated.

The reporter cell line for VZV was developed based on the specific activation of the viral promoter [48]. Several promoter sequences of VZV have been tested, and the best sequence was shown to be the ORF9 promoter because of its specific, strong activation. The luciferase gene is located downstream of the ORF9 promoter. After plasmid assembly, MeWo cells and BSC40 cells were transfected, and stable transformants were selected by using G418. One of the obtained cell clones was employed for further research, as it showed high luciferase activity that appeared in a dose dependent manner in response to VZV and a low background activity level in the absence of VZV. Additionally, the activation of the reporter occurred in the case of cocultivation of infected cells and uninfected cells with a reporter cassette. To detect nonspecific reporter reactivation, cell lines were infected by HCMV, herpesvirus 6 (Z29) [106], and human herpesvirus 7 (SB) [107]. In all of these cases, no luciferase activity was revealed. However, the infection of the indicator cells by HSV1 activated luciferase activity to a similar level to that observed during the 24 h after VZV infection. However, HSV1-infected cells exhibited a strong cytopathic effect at 48 h after infection; thus, luciferase activity decreased, making it feasible to differentiate HSV1 infection from VZV infection.

## 4. Conclusions

Virus detection methods include a range of molecular and cellular approaches. Cell-based methods account for a large portion of virology research. Any cell line used for virus detection must be characterized by sensitivity, which refers to both susceptibility and permissivity. In general, sensitivity is the ability of the cell to be infected by a small amount of the virus independent of the species. In the laboratory, permissivity can be achieved by knocking out cell antiviral response genes or by the simple selection of sensitive cell lines among different cultures from a laboratory collection. Susceptibility is a property of the cell line to be infected by certain virus species. In the laboratory, susceptibility can be increased by increasing the expression of virus-binding cell receptors. The improvement of these two properties makes cell lines a convenient tool for virus isolation and study. The study of virus infection has traditionally been carried out on the basis of CPE, but this approach does not work for every virus, and additional time-consuming studies are necessary in such cases. This problem has been solved by the use of cell lines carrying molecular sensor systems that allow the detection of infectious virus agents by the presence of easily detected reporter proteins. Such an approach allows the differentiation of a specific virus species or family. This method can be used for quantitative studies as a faster analog of CPE approaches. The optimization of cell-based methods in virology can be achieved by integrating molecular techniques to obtain increased sensitivity in molecular reporter construction.

## Figures and Tables

**Table 1:** Current approaches for virus detection and characterization.

The target of detection and application		Methods	Advantages	Limitations
**Viral proteins and nucleic acids detection**	Rapid known virus detection with known invariable portions of the genome	Amplification-based [1]	Fast, cheap, highly sensitive detection	Detection of the presence/absence of a viral nucleic acid; false-positive results, difficulty detecting viruses with highly variable genomes; detection of only known viruses, requires knowledge of the nucleotide sequence; inability to differentiate between infectious and noninfectious viruses
		Methods related to CRISPR/Cas use [2,3]	Can be used in the field, relatively short time to obtain results, high sensitivity	Detection of the presence/absence of a viral nucleic acid; detection of only known viruses, requires knowledge of the nucleotide sequence
		Southern/northern hybridization	Sensitive detection. Many types of specimens can be used (Blood, cerebrospinal fluid, urine, bronchoalveolar lavage, etc.)	Requires knowledge of the virus nucleotide sequence
	Searching for new viruses	NGS [4]	Determination of the nucleotide sequence of viruses	Difficulty of identifying RNA viruses in patient samples due to additional stages of sample preparation and, as a consequence, a decrease in the viral RNA to host RNA ratio. Difficulties in data processing
	Rapid detection of known viruses with known viral proteins	Immunoassays [5] (direct determination of viral proteins and indirect determination of IgG and IgM)	For proteins, the ability to detect previous exposure.	Likelihood of false-positive results; possible cross-reactivity with closely related viruses
Detection of viable viral particles	Extraction and study of live viral particles	Cell-based approach with CPE detection [6]	Determination of live virus particles in clinical material, studying their pathogenicity and transmission mechanisms. Detection of viruses with a highly variable genome that cannot be determined by PCR methods.	The main problem is the long time period (up to several weeks) required for a result to be available. Cell cultures are also very susceptible to bacterial contamination and toxic substances in the clinical virus specimen. Additionally, many viruses will not grow in cell culture (Epstein-Barr virus, hepatitis B, hepatitis C, parvovirus, etc.)
		Cell-based reporter [7,8]		It is necessary to develop different approaches to study specific viruses. Suitable for viruses with annotated genomes.
